# An update on targeted therapies in systemic sclerosis based on a systematic review from the last 3 years

**DOI:** 10.1186/s13075-021-02536-5

**Published:** 2021-06-01

**Authors:** Corrado Campochiaro, Yannick Allanore

**Affiliations:** 1 Unit of Immunology, Rheumatology, Allergy and Rare Diseases (UnIRAR) IRCCS San Raffaele Hospital, Vita-Salute San Raffaele University, Via Olgettina 60, 20132 Milan, Italy; 2 Service de Rhumatologie, Hôpital Cochin, Université de Paris, 27 rue du Faubourg Saint-Jacques, 75014 Paris, France

**Keywords:** Systemic Sclerosis, Targeted therapy, Interstitial lung disease, Clinical Trial, Observational study, Systematic review

## Abstract

New molecular mechanisms that can be targeted with specific drugs have recently emerged for the treatment of systemic sclerosis (SSc) patients. Over the past 3 years, the achievement of one large phase 3 trial has led to the approval by drug agencies of the first drug licenced for SSc-related interstitial lung disease. Given this exciting time in the SSc field, we aimed to perform a systemic literature review of phase 1, phase 2 and phase 3 clinical trials and large observational studies about targeted therapies in SSc. We searched MEDLINE/PubMed, EMBASE, and ClinicalTrials.gov for clinical studies from 2016 with targeted therapies as the primary treatment in patients with SSc for skin or lung involvement as the primary clinical outcome measure. Details on the study characteristics, the trial drug used, the molecular target engaged by the trial drug, the inclusion criteria of the study, the treatment dose, the possibility of concomitant immunosuppression, the endpoints of the study, the duration of the study and the results obtained were reviewed. Of the 973 references identified, 21 (4 conference abstracts and 17 articles) were included in the systematic review. A total of 15 phase 1/phase 2 clinical trials, 2 phase 3 clinical trials and 2 observation studies were analysed. The drugs studied in phase 1/phase 2 studies included the following: inebilizumab, dabigatran, C-82, pomalidomide, rilonacept, romilkimab, tocilizumab, tofacitinib, pirfenidone, lenabasum, abatacept, belimumab, riociguat, SAR100842 and lanifibranor. All but 3 studies were performed in early diffuse SSc patients with different inclusion criteria, while 3 studies were performed in SSc patients with interstitial lung disease (ILD). Phase 3 clinical trials investigated nintedanib and tocilizumab. Nintedanib was investigated in SSc-ILD patients whereas tocilizumab focused on early diffuse SSc patients with inflammatory features. Two observational studies including > 50 patients with rituximab as the targeted drug were also evaluated. All these studies offer a real hope for SSc patients. The future challenges will be to customize patient-specific therapeutics with the goal to develop precision medicine for SSc.

## Introduction

**Fig. 1:**
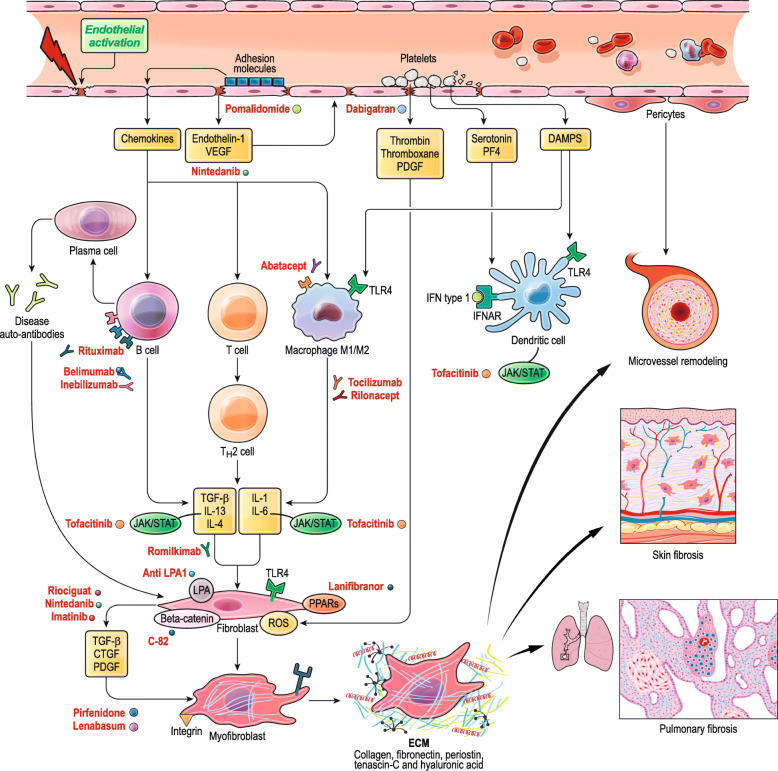
The pathogenesis of systemic sclerosis. The highly specific mesenchymal cell activation and related fibrosis underlying systemic sclerosis are thought to be induced by vascular injury and endothelial activation leading to an uncontrolled inflammatory/immune reaction. The main actors and players are indicated in the cartoon together with the targets of recently performed clinical trials. VEGF = vascular-endothelial growth factor. PF4 = platelet-factor 4. DAMPS = damage-associated molecular patterns. TLR4 = toll-like receptor 4. IFNAR = interferon receptor. JAK = Janus kinase. PPAR = peroxisome proliferator-activated receptor. LPA = lysophosphatidic acid receptor. ROS = reactive oxygen species. TGF = tissue growth factor. CTGF = connective tissue growth factor. PDGF = platelet-derived growth factor. ECM = extracellular matrix

Systemic sclerosis (SSc) is an orphan multiorgan connective tissue disease characterized by microangiopathy, immune dysregulation and fibrotic changes affecting the skin and internal organs [1, 2]. Although the pathogenesis of SSc is far from being fully understood, multiple pathogenic mechanisms and different cell types have been implicated in the disease process [3]. Chronic vascular injury, endothelial activation and immune activation are all thought to be crucial for secondarily fibroblast activation and related fibrogenesis [4]. The release of different soluble mediators including endothelin-1, chemokines and growth factors together with an increased expression of adhesion molecules and platelet activation can lead to the recruitment and activation of immune inflammatory cells, including type 2 helper (Th2) T cells that secrete transforming growth factor-β (TGFβ), interleukin 13 (IL-13) and IL-4 known to promote fibrogenesis; B cells that produce autoantibodies and IL-6; macrophages that release TGFβ, IL-1 and IL-6 and dendritic cells that secrete type 1 interferon (IFN) [4]. Furthermore, activated platelet can increase the production of platelet-derived growth factor (PDGF), thrombin, thromboxane, serotonin and platelet factor 4 (PF4). All these mediators contribute to the phenotypic differentiation of fibroblasts into myofibroblasts which are responsible for the generation of reactive oxygen species (ROS) and other fundamental release of growth factors that include TGFβ, connective tissue growth factor (CCN2) and PDGF. Regulatory pathways activated by lipid mediators and intracellular molecules can further modulate extracellular matrix (ECM) production (see Fig. 1). In this review, we will go through the recent evidences obtained in phase 1, phase 2 and phase 3 clinical trials and large observational studies about targeted therapies in SSc over the last 3 years.

## Methods

The study protocol was developed according to the Preferred Reporting Items for Systematic Reviews and Meta-Analyses (PRISMA) guidelines. Eligibility criteria are as follows: phase 1, phase 2, phase 3 or observational studies reporting the use of targeted therapies in the treatment of SSc patients for skin or lung involvement. Applying the PICOs framework, we evaluated publications that fulfilled the following study characteristics:

1. Participants: Adult (≥ 18 years old) patients with a diagnosis of systemic sclerosis
2. Interventions: Studies reporting the outcome of targeted therapies for lung or skin involvement in SSc patients.
3. Comparison: Where applicable, comparison of lung or skin outcomes in the group of SSc patients treated with the targeted therapy versus control group was made.
4. Outcomes: Effectiveness of targeted therapies for lung or skin involvement in SSc patients. Both primary and secondary efficacy endpoints and safety endpoints were included.
5. Study design: (i) phase 1, phase 2 or phase 3 trials, (ii) observational studies including ≥ 50 SSc patents with targeted therapies as the primary treatment in patients with SSc for skin or lung involvement, (iii) articles published in English, (iv) articles published from January 2016 to July 2020. Post hoc analyses of clinical trials were excluded.

Information source and search criteria are as follows: A literature search of MEDLINE/PubMed, ClinicalTrials.gov and EMBASE databases was performed. The following search criteria were used: ((systemic sclerosis) OR (scleroderma)) AND ((phase 1) OR (phase 2) OR (phase 3) OR (trial) OR (observational)).

For study selection, abstracts’ titles were screened independently by both reviewers (CC and YA) for relevance and eligibility of studies for full text review. Divergences in agreement were resolved through discussion at each step of the study selection process.

For data extraction, data was extracted by CC and reviewed by YA. The data extraction form included the following details about the studies: date of publication, study population and intervention characteristics, the trial drug used, the molecular target engaged by the trial drug, the inclusion criteria of the study, the treatment dose, the possibility of concomitant immunosuppression, the endpoints of the study, the duration of the study, adverse effects and outcomes.

For risk of bias assessment, since some of the study included were not randomized, risk of bias was assessed for these studies using the Risk of Bias Assessment Tool for Non-randomized Studies (RoBANS). Studies’ risk of bias was rated as “high”, “low” or “unclear” on each of the dimensions (selection, performance, detection, attrition and reporting).

## Results

**Fig. 2:**
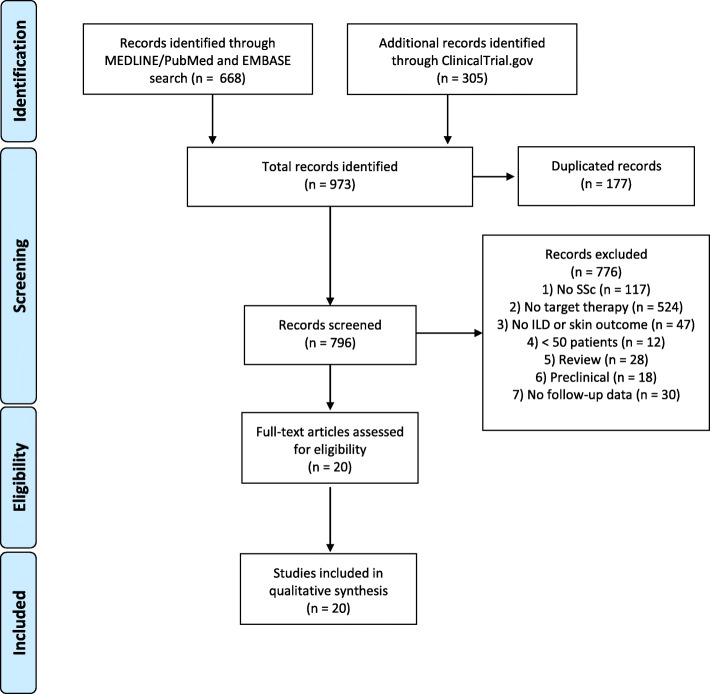
Flowchart summarizing the study selection process for systematic literature review

Searches of MEDLINE/PubMed and EMBASE (668 records) and ClinicalTrial.gov (305 records) were undertaken identifying a total of 973 records. After removal of duplications (n = 177), the remaining 796 articles were screened for eligibility during a title and abstract review undertaken by both reviewers. A total of 20 studies fulfilled inclusion criteria including 4 conference abstracts and 16 full articles. No studies were excluded due to language. There was complete agreement between the reviewers for studies’ eligibility for full text review. A total of 16 phase 1/phase 2 clinical trials, 2 phase 3 clinical trials and 2 observation studies were analysed. An overview of the study selection process is summarized in Fig. 2.

### Phase 1-2 trials

#### Inebilizumab

CD19 is critically involved in establishing intrinsic B cell signalling thresholds through modulating both B cell receptor-dependent and independent signalling; it plays a critical role in maintaining the balance between humoral, antigen-induced response and tolerance induction [5]. Inebilizumab (MEDI-551) is an anti-CD19 monoclonal antibody that leads to antibody-dependent, cell-mediated cytotoxicity of B cells [6]. A phase I, randomized, placebo-controlled, escalating, single-dose study was performed in SSc patients (both limited and diffuse cutaneous) [7]. Twenty-eight patients were enrolled, 24 of these received a single dose of inebilizumab of 0.1–10 mg/kg. The vast majority (96%) of patients treated with inebilizumab experienced treatment-emergent adverse events (compared to 75% of placebo patients), the most common being nausea (17%) and fatigue (17%). Drug-related side effects were classified as mild and 4 infusion-related reactions were observed. Only two serious adverse events were recorded in the inebilizumab group: supraventricular tachycardia and subclavian vein thrombosis. A potential effect of inebilizumab on skin thickness but not on pulmonary function tests was observed as the mean modified Rodnan skin score (mRSS) change from baseline to day 85 in the inebilizumab group was − 5.4 ± 4.2 compared to 2.3 ± 6.1 in the placebo group. No clear relationship was found with the drug dose. Conversely, circulating B cell depletion was observed in a dose-dependent fashion.

#### Dabigatran

Coagulation was originally thought to be an acute and transient response to tissue injury, responsible primarily for promoting haemostasis by initiating the formation of fibrin plugs to enmesh activated platelets within the walls of damaged blood vessels. However, there is now mounting evidence that coagulation plays a critical role in orchestrating inflammatory and fibroproliferative responses during wound healing, as well as in a range of pathological contexts across several organ systems [8]. Dabigatran, a direct thrombin inhibitor, was shown to attenuate organ fibrosis in a mouse model of SSc [9]. Moreover, thrombin was also demonstrated to stimulate fibroblast proliferation and myofibroblast transition [10]. Dabigatran has been studied in a 6-month, phase 1, prospective, single-centre, open-label study in SSc patients with interstitial lung disease (ILD) [11]. The dose was 75 mg twice daily. Due to the increased risk a bleeding, patients with history of gastrointestinal haemorrhage or gastric antral vascular ectasia were excluded. Exploratory endpoints included patient-reported outcomes, pulmonary function tests and mRSS at months 3 and 6. A total of 15 patients were enrolled. Over the study period, no serious adverse event was observed and dabigatran was well-tolerated. A significant improvement in the mRSS was observed (− 6.6 ± 6.4, p = 0.002), but no significant modification in the lung function tests (%FVC predicted, %FEV1 predicted and %DLCO predicted) was recorded.

#### C-82

Several lines of evidence suggest that Wnt signalling is implicated in SSc skin fibrosis [12]. C-82 blocks the interaction between β-catenin and the transcriptional co-activator and acetyltransferase proteins CBP and p300 [13], leading to the inhibition of Wnt-activated genes. A placebo-controlled, double-blinded clinical trial in patients with early (median disease duration 8 months) dcSSc was performed with daily C-82 topical formulation for 4 weeks [14]. Although no clinical effect (mRSS including the local skin score on each forearm at baseline and after 4 weeks of treatment) was observed over the study period, repeated skin biopsies demonstrated a weak downregulation of THBS1 and COMP and an upregulation of two clusters of genes in subcutaneous fat cells that negatively correlate with the severity of skin involvement in SSc. The authors concluded that, as suggested by gene expression analysis, longer treatment with topical C-82 might promote fat regeneration in SSc skin.

#### Pomalidomide

Pomalidomide (POM) is an anti-angiogenic and immunomodulatory molecule similar to thalidomide. POM binds to cereblon which is responsible for Ikaros and Aiolos degradation and eventually leads to immune-modulation of myeloid and lymphoid cells [15]. A previous open-label, dose-escalating, 12-week study had shown a beneficial effect of thalidomide in skin fibrosis and marginally in gastroesophageal reflux symptoms and digital ulcer healing in 11 SSc patients [16]. Furthermore, biopsies from SSc patients treated with thalidomide had suggested a pro-Th1 immunomodulatory effect for thalidomide. Given this background, a Phase 2, multicentre, randomized, double-blind, placebo-controlled, parallel-group study was conducted in SSc patients with ILD [17]. Fifty nine patients were screened and twenty-three SSc patients were randomized 1:1 to POM 1 mg once daily or placebo for 52 weeks of blinded treatment and a 2-year open-label extension phase. The endpoints of the study were the changes in % predicted forced vital capacity (%pFCV), mRSS, and gastrointestinal (GI) symptomatology evaluated through the UCLA Scleroderma Clinical Trials Consortium Gastrointestinal Tract (SCTC GIT 2.0) questionnaire. Unfortunately, although POM was generally well-tolerated, subjects’ enrolment was discontinued early because of insufficient recruitment (original targeted sample size was 88 patients) and an interim analysis showed no statistically significant improvement in any of the 3 coprimary efficacy endpoints (changes from baseline in FVC, mRSS, or UCLA SCTC GIT 2.0 at week 24 or week 52). Unfortunately, no clear conclusions could be drawn because too few subjects were enrolled.

#### Rilonacept

Rilonacept is a dimeric fusion protein consisting of the human interleukin-1 receptor component (IL-1R1) and IL-1 receptor accessory protein (IL-1RAcP), it is also known as “IL-1 Trap” and it binds and neutralizes IL-1β thus preventing IL-1 from binding with IL-1 cell surface receptors. Rilonacept also binds IL-1alpha and IL-1 receptor antagonist but with reduced affinity [18]. Although the exact mechanism is still uncertain, IL-1 family and inflammasome activation have been implicated in murine models of fibrosis [19]. For these reasons, a phase I/II biomarker, randomized, double-blind, placebo-controlled trial of rilonacept was performed in SSc patients [20]. The primary endpoint was the level of skin expression of the 2G SSc gene biomarkers (THBS1 and MS4A4) as surrogate for the mRSS, while the secondary endpoint was the change in the mRSS. Nineteen patients were randomized 2:1 rilonacept 320 mg loading dose at day 0 ad then 160 mg weekly versus placebo. Skin biopsies were obtained before rilonacept treatment initiation and at week 7. Both the primary and the secondary endpoints were not met in this short-term trial as after 6 weeks no modification in gene expression or in the mRSS between treated and placebo patients were observed.

#### Romilkimab

Romilkimab is a bispecific monoclonal antibody that binds and neutralizes both IL-4 and IL-13 [21]. These are Th2-derived cytokines that have been found to be elevated both in the serum and in the skin biopsies of SSc patients and have also been implicated in the fibrotic pathway of SSc [22]. Moreover, mice with genetic deletion of IL-13 are protected from fibrosis [22]. A phase 2A, randomized, double-blind, placebo-controlled, 24-week trial was performed in dcSSc patients [23]. Ninety-seven early (disease duration ≤ 36 months) dcSSc patients, with or without background immunosuppressive therapy, were randomized 1:1 to romilkimab 200 mg sc or placebo. The primary endpoint of the study was the mean change in mRSS, secondary endpoints were FVC/DLCO and Health Assessment Questionnaire – Disability Index (HAQ-DI) questionnaire. After 24 weeks, patients treated with romilkimab showed a statistically significant (one-sided p value = 0.029) improvement in the mRSS (− 4.76 ± 0.86 versus − 2.45 ± 0.85 in the placebo group). A subgroup analysis suggested also an additive effect between background immunosuppressive therapy and romilkimab. While no secondary endpoint was met (romilkimab was associated with a reduced decline in FVC), exploratory endpoints suggested a possible effect of romilkimab on overall pain, Raynaud’s phenomenon, digital ulcers and quality of life (EQ-5D-5L questionnaire). Side effects were similar in the two groups and 1 death occurred in both arms due to SSc-related complications.

#### Tocilizumab

Tocilizumab (TCZ) is an interleukin 6 receptor-inhibitor. IL-6 has been deeply implicated in the pathogenesis of SSc. Indeed, IL-6 has major role in both B and T cell differentiation and fibroblasts transformation into activated myofibroblasts which are fundamental for extracellular matrix production [24]. Importantly, in the bleomycin-induced SSc mouse model, IL-6 blockade was associated with improvement in skin fibrosis and reduction in α smooth-muscle actin protein expression and myofibroblast counts [25]. In vivo studies have demonstrated high IL-6 concentrations in the sera and skin biopsies of SSc patients and its levels are associated with more severe disease activity and disease progression together with reduced life expectancy [26]. Moreover, IL-6 levels can predict the extent of skin involvement in early SSc patients [27]. The results of a phase 2, randomized, double-blind, placebo-controlled trial investigating the safety and efficacy of subcutaneous TCZ in adults SSc patients (faSScinate trial) were published in 2016 [28]. Patients with early disease (disease duration ≤ 5 years) and enriched for inflammatory phase were assigned with a 1:1 ratio to TCZ 162 mg sc weekly or placebo. Background immunosuppressant was not allowed. The primary endpoint was the mean change in the mRSS at 24 weeks. Eighty-seven patients were enrolled. Although the primary endpoint was not met, the mean mRSS reduction favoured the TCZ group (− 2.70, 95% CI − 5.85 to 0.45; p = 0.0915). A further and almost significant improvement was observed at 48 weeks in TCZ-treated patients compared to placebo (− 3.55, 95% CI − 7.23 to 0.12; p = 0.0579). Moreover, a significantly smaller decrease in FVC in the tocilizumab group compared to placebo was observed at 24 weeks (TCZ − 34 mL versus PBO − 171 mL; p = 0.0368). No significant differences were observed in disability, fatigue, itching or patient’ or clinician’s global disease severity. Although the incidence of serious adverse events was similar between the two groups (33% vs 34%), serious infections were more common in the TCZ group (16% vs 5%) and one patient in the TCZ group died. These encouraging results paved the way to the phase 3 trial of tocilizumab in SSc (focuSSced trial) whose results are discussed in the “Phase 3 Trials” paragraph.

#### Tofacitinib

The JAK/STAT pathway is the principal signalling mechanism for several cytokines and growth factors [29]. STAT3 is part of the JAK/STAT pathway and has a critical role in skin and lung fibrosis [30]. Tofacitinib is a “*pan* JAK inhibitor” as it has a low JAK selectivity being able to block JAK1, JAK2, and JAK3 [31]. Some major mediators which are deemed fundamental in SSc pathogenesis are indeed involved in JAK/STAT signalling pathway: IL-6, IFN type 1 and 2 and most importantly IL-4 and IL-13 [32]. Moreover, different mouse models of SSc showed a potent anti-fibrotic effect for tofacitinib [30]. The safety and efficacy of tofacitinib in SSc was recently tested in a phase I/II, 6-month, double-blind, randomized placebo-controlled trial conducted in early (≤ 60 months) dcSSc patients [33]. Tofacitinib was used at a dose of 5 mg twice a day and stable background immunosuppressive therapies were allowed. The primary outcome was the proportion of patients who experienced ≥ Grade 3 adverse events. Secondary endpoints were the mRSS at month 6, HAQ-DI, patient and physician global assessments, and the ACR composite measure: Combined Response Index in Systemic Sclerosis (CRISS). Fifteen patients were randomized 2:1. Thirteen patients were on stable daily dose of immunosuppressive drugs (12 on mycophenolate mofetil (MMF) and 1 on methotrexate (MTX)). Over the study period, tofacitinib was well tolerated with no patient experiencing ≥ Grade 3 adverse events. A trend towards improvement of clinical outcome measures was observed. This preliminary study supports further evaluation of tofacitinib in SSc.

#### Pirfenidone

Pirfenidone is a synthetic anti-fibrotic agent already approved by the FDA for the treatment of patients with idiopathic pulmonary fibrosis [34]. In vitro studies showed that pirfenidone inhibits myofibroblast differentiation and blocks TGF-β and STAT-3 activation [35]. The safety and efficacy of pirfenidone was evaluated in an open-label, 16-week, phase II trial in SSc-ILD patients randomized 1:1 to either a 2- or 4-week pirfenidone titration starting at 801 mg daily and titrating up to 2403 mg daily maintenance dose (LOTUSS trial) or placebo [36]. Concomitant background immunosuppressive therapy was allowed. Eligibility criteria included disease duration ≤ 7 years, %predicted FVC > 50% and DLCO > 40%. The primary endpoint was the assessment of adverse events, secondary endpoints were the change in %predicted FVC and DLCO, mRSS and patient-reported outcomes (Mahler baseline and Transition Dyspnoea Indices, HAQ-DI and patient’s global assessment). Sixty-three patients were enrolled and the vast majority (96.8%) experienced adverse events especially during the titration period. The most common adverse events were nausea, headache and fatigue and were reported regardless of the titration schedule. Notably, more patients in the 2-week titration group discontinued the treatment compared to patients treated with the 4-week titration scheme. 63.5% of patients were on MMF therapy but its concomitant use did not affect tolerability. No change in disease outcomes was observed. In conclusion, while pirfenidone was globally well tolerated in SSc-ILD patients especially in patients treated with 4-week titration scheme no conclusion of its efficacy could be drawn. This study paved the way to the ongoing Scleroderma Lung Study III where pirfenidone is used in combination with MMF (clinicaltrials.gov NCT03221257).

#### Lenabasum

Lenabasum is a selective type 2 cannabinoid receptor agonist [37]. Cannabinoid 2 receptors are mainly expressed on immune cells and tissue-resident stromal cells and their activation has been demonstrated to reduce inflammation and tissue fibrosis with only minimal psychoactive effects [38]. A double-blind, randomized, placebo-controlled, 16-week, Phase 2 trial was performed in early (< 6 years disease duration) dcSSc patients [39]. Forty-two patients were enrolled and they were allowed to remain on stable background immunosuppression. Patients were treated with the following scheme: 5 mg/day, 20 mg/day or 20 mg bis in die for 4 weeks and then 20 mg bis in die for 8 weeks. The primary endpoint was CRISS scores. No serious or severe adverse events related to lenabasum were observed. Adverse events that occurred in more than 10% of subjects during 16 weeks with either placebo or lenabasum (% pbo vs % lenabasum) were dizziness (13 vs 22%), fatigue (7 vs 19%), headache (7 vs 11%), upper respiratory tract infection (0 vs 11%).

At week 16, patients treated with lenabasum (merge of the 2 treated groups) had a significant improvement in CRISS scores compared to placebo patients (p = 0.044). No dosing effect was observed. Skin biopsies were also taken and they showed a reduction in key genes implicated in inflammation and fibrosis only in lenabasum-treated patient. The trial was followed by a long-term open-label safety and efficacy study [40]. Patients who had completed the 16-week Phase 2 study were enrolled to continue with lenabasum 20 mg twice a day. Thirty-six patients were enrolled and 26 patients were treated for > 92 weeks. At week 92, the vast majority of patients experienced at least 1 adverse event classified as mild or moderate. Notably, only in 7 (19%) patients the adverse event was considered related to lenabasum (fatigue, mild disturbances in attention and mild lethargy). The long-term study lenabasum supported its efficacy as improvements were observed in CRISS scores (median score 0.96), mRSS (mean decline 10.3 from baseline), HAQ-DI, physician global assessment and itch. %predicted FVC values declined by 3.2% from study start, but the trial design of this open period limits the conclusion. In conclusion, lenabasum has shown an acceptable safety and tolerability profile and its potential efficacy in several endpoints, although several methodological limitations have emerged (merge of the doses, short-term study, modest sample size). Unfortunately, while the results of the phase 3 double-blind, randomized, placebo-controlled study assessing the efficacy and safety of lenabasum in dcSSc (RESOLVE-1) have not yet been published, a press release in September 2020 stated that the study did not meet the primary endpoint (CRISS).

#### Abatacept

Abatacept is recombinant fusion protein that binds to CD80 and CD86 thus preventing T cell co-stimulation by CD28 [41]. A 12-month, investigator-initiated, multicentre, double-blind, randomized, placebo-controlled phase 2 study was performed in early (disease duration < 36 months) dcSSc patients [42]. Patients were randomized 1:1 to abatacept 125 mg weekly subcutaneous or placebo. No background immunosuppression was allowed. The primary endpoints were as follows: modification in the mRSS and safety. Eighty-eight patients were enrolled. At 12 months, although a trend of efficacy could be observed, no significant difference in the mRSS was measured (− 6.24 in the abatacept group compared to − 4.49 in the PBO group, p = 0.28), whereas HAQ-DI and some other composite measures significantly favoured abatacept-treated patients. Inflammatory gene expression significantly declined in patients treated with abatacept and the safety profile was satisfactory. Over the 6-month open-label extension, no new safety signals emerged. Moreover, clinically meaningful improvement in the mRSS was observed in both the abatacept and PBO groups when patients transitioned to abatacept supporting further studies of abatacept in dcSSc [43].

#### Belimumab

Blys is a cytokine expressed in B cell lineage cells that acts as a potent B cell activator. It has been shown to play an important role in the proliferation and differentiation of B cells but it could also act on some innate immune cells like monocytes [44]. Belimumab is a recombinant antibody that binds to and inhibits soluble human BLys. Its biological activity causes mainly apoptosis of B cells and decreases autoantibody production [45]. A 52-week, investigator-initiated, single-centre, double-blind, placebo-controlled, pilot study was performed in early (disease duration < 3 years) dcSSc patients recently started on mycophenolate mofetil [46]. Twenty patients were enrolled and randomized 1:1 to belimumab 10 mg/kg intravenously at a 2-week interval for the first three doses and then at 4-week intervals until week 48 while also on mycophenolate mofetil therapy (1 g twice a day). At 52 weeks, no significant reduction in the mRSS was observed between the belimumab and the placebo group (median reduction − 10 and − 3 respectively, p = 0.411). No significant differences in the number of adverse events between the two groups were observed. Of note, a significant decrease in skin B cell signalling and profibrotic gene expression was observed in patients treated with belimumab.

#### Riociguat

Riociguat is soluble guanylate cyclase (sGC) stimulator with potential anti-fibrotic effects and proved efficacy in patients with pulmonary arterial hypertension associated with connective tissue diseases [47]. A 52 weeks, double-blind, placebo-controlled, multi-centre, randomized phase 2 study was undertaken in early (disease duration ≤ 18 months) dcSSc patients to investigate the potential effects on skin invovlement [48]. No background immunosuppression was allowed. In total, 121 patients were randomized 1:1 to either riociguat 0.5 mg (up-titrated to a maximum dose of 2.5 mg three times a day over 10 weeks). The primary endpoint of the study was the change in mRSS. Secondary endpoints included ACR CRISS, HAQ-DI, mRSS progression rate and change in %predicted FVC. At 52 weeks, the primary endpoint was not met as the mean mRSS was not statistically different between the two groups: mean mRSS was 14.63 ± 6.56 for riociguat vs 15.73 ± 10.48 for placebo (least squares mean treatment difference − 2.34 [95% CI − 4.99, 0.30; p = 0.08]). Among the secondary endpoints, only the difference in mRSS progression rate showed a significant positive effect for riociguat patients (− 18%, p = 0.02). No significant adverse events were observed in the riociguat group.

#### SAR100842

SAR100842 is a selective oral antagonist of the lysophosphatidic acid receptor 1 (LPA1). Given its biological activity in stimulating mesenchymal cell migration and extracellular matrix production, LPA1 has been suggested to be implicated in the pathogenesis of SSc [49]. An 8-week, double-blind, randomized, placebo-controlled study followed by a 16-week open-label extension was performed in early (disease duration < 36 months) dcSSc patients [50]. Patients could be on stable background immunosuppressive therapy. The primary endpoint was the safety and tolerability of SAR100842, while exploratory endpoints included gene signature in patients’ skin biopsies. Seventeen patients were enrolled to receive either SAR100842 300 mg twice a day or placebo. At week 8, the most common adverse events in SAR100842 patients were headache, diarrhoea and nausea. The mRSS reduction at week 8 was higher in the SAR100842 group compared to the placebo group but it was not statistically different (− 3.6 versus − 2.8, p = 0.46). LPA-related gene analysis in skin biopsies confirmed LPA1 target engagement.

#### Lanifibranor

Lanifibranor is a small molecule that activates all 3 PPAR isoforms. In preclinical SSc models, it was shown to reduce skin and lung fibrosis [51]. A phase 2 trial of lanifibranor (FASST study) has been performed in early (disease duration < 36 months) dcSSc patients. Background immunosuppression was allowed. The results of the trial have not been published yet but the preliminary results were press-released. A total of 145 patients were enrolled: 48 patients were treated with lanifibranor 1200 mg daily, 49 patients with lanifibranor 800 mg daily and 48 with PBO. At 48 weeks, no significant change in the mRSS was observed among the three groups (− 3.7 in the 800 mg group, − 4.3 in the 1200 mg group and − 4.9 in the placebo group). Lanifibranor was associated with a good safety profile with only one patient experiencing peripheral oedema in the 1200 mg group.

Table 1 summarizes Phase 1–2 trials.

**Table 1:** Targeted therapies of Phase 1 and Phase 2 studies in SSc patients

Trial drug	Target	Inclusion criteria	Treatment	IS	Endpoints	Duration	Results
Inebilizumab	B cells (CD19)	Localized mRSS ≥ 2	Single dose 0.1–10 mg/kg	Yes	Safety Tolerability	12 weeks	Safe and well-tolerated
Dabigatran	Thrombin	SSc-ILD HRCT ≥ 20% FVC < 70% Early (≤ 10 years)	75 mg twice a day	Yes	Safety Tolerability	6 months	Safe and well-tolerated
C-82	β-Catenin Signaling	Early (median 8 months) dcSSc (mRSS ≥ 12) Localized mRSS ≥ 2	Topical formulation	Yes	AE Gene biomarkers	4 weeks	Well-tolerated Weak genes downregulation
Pomalidomide	Angiogenesis immunosuppression	SSc-ILD FVC > 45 > 70 FVC > 70, recent loss of 5% HRCT > 20% Early (< 7 years)	1 mg/day	No	%pFVC mRSS SCTC GIT 2.0	52 weeks	Negative
Rilonacept	IL-1	Early (< 24 months) dcSSc (mRSS ≥ 15)	320 mg sc loading dose 160 mg sc weekly	No	Change in expression in 2G SSc genes mRSS	6 weeks	Negative
Romilkimab	IL-4 and IL-13	Early (≤ 36 months) dcSSc (mRSS ≥ 10)	200 mg sc weekly	Yes	mRSS FVC/DLCO HAQ-DI	24 weeks	mRSS difference − 2.31 (p = 0.029) in favour of Romilkimab
Tocilizumab	IL-6	Early (< 5 years) dcSSc (mRSS ≥ 10)	162 mg sc weekly	No	mRSS FVC	48 weeks (primary outcome at 24 weeks)	mRSS change favoured TCZ (p = 0.058) Smaller decrease in FVC in TCZ
Tofacitinib	JAK1 and 3	Early (< 5 years) dcSSc (mRSS ≥ 10)	5 mg twice a day	Yes	Grade ≥ 3 AE mRSS HAQ-DI CRISS	24 weeks	No Grade 3 AE Improvement trend
Pirfenidone	Myofibroblast TGF-β STAT-3	SSc-ILD FVC ≥ 50% DLCO ≥ 40% Early (< 7 years)	2- or 4-week titration 801 mg daily to 2403 mg daily	Yes	AE FVC and DLCO PRO	16 weeks	4-week titration better tolerated No change
Lenabasum	Cannabinoid receptor 2	Early (< 3 years or > 3 years and < 6 years with CRP > 3) dcSSc (ΔmRSS ≥ 5 last 6 months, total mRSS ≥ 12)	5 mg/day, 20 mg/day or 20 mg twice a day for 4 weeks and then 20 mg twice a day for 8 weeks.	Yes	CRISS	16 weeks	Improvement (p = 0.044) in mRSS PRO PGA HAQ-DI
Abatacept	B/T cells interaction (CD80/CD86)	Early dcSSc (≤ 18 months, mRSS ≥ 10; > 18 and ≤ 36 months, mRSS ≥ 15)	125 mg sc weekly	No	mRSS Safety	12 months	Negative Good safety profile
Belimumab	BLys	Early (≤ 3 years) dcSSc (mRSS > 15) recently started on MMF (2 g)	10 mg/kg iv 2-weekly for the first three doses and then 4-weekly	Yes	mRSS Safety Tolerability	52 weeks	No significant mRSS change Safe and well-tolerated
Riociguat	Guanylate Cyclase	Early (≤ 18 months) dcSSc (mRSS ≥ 10)	0.5 mg (up-titrated to a maximum dose of 2.5 mg three times a day)	No	mRSS CRISS HAQ-DI FVC	52 weeks	Negative Reduced mRSS progression in Riociguat
SAR100842	Lysophosphatidic acid receptor 1	Early (≤ 36 months) dcSSc (mRSS ≥ 15)	300 mg twice a day	Yes	Safety Tolerability mRSS	24 weeks	Safe and well-tolerated No significant change in mRSS
Lanifibranor	PPAR	Early (≤ 3 years) dcSSc (mRSS ≥ 10)	400 mg twice a day 600 mg twice a day	Yes	mRSS FVC and DLCO CRISS and PRO	48 weeks	No significant change in mRSS

*IS* immunosuppressive treatment, *mRSS* modified Rodnan skin score, *SSc* systemic sclerosis, *ILD* interstitial lung disease, *HRCT* high-resolution computed tomography, *FVC* forced vital capacity, *AE* adverse events, *SCTC GIT* Scleroderma Clinical Trials Consortium Gastrointestinal Tract, *DLCO* diffusing lung capacity for carbon monoxide, *HAQ-DI* Health Assessment Questionnaire – Disability Index, *CRISS* Combined Response Index in Systemic Sclerosis, *PRO* patient-reported outcome

### Phase 3 trials

#### Nintedanib

Nintedanib is a multi-tyrosine kinase inhibitor which blocks FGF receptor-1, VEGF receptor-2 and PDGF receptor-α and β [52]. Its anti-fibrotic and anti-inflammatory activity was already demonstrated in preclinical models of SSc-ILD [53]. It is approved for the treatment of idiopathic pulmonary fibrosis [54]. A randomized, double-blind, placebo-controlled trial was performed in patients with SSc-ILD. Patients with early disease (< 7 years), regardless of their disease subset, but with a high-resolution computed tomography showing ≥ 10% ILD were enrolled in a 1:1 ratio to either oral nintedanib 150 mg twice a day or placebo (SENSCIS trial) [55]. Patients were allowed to be on stable background immunosuppression. The primary endpoint of the study was the annual rate of decline in FVC assessed. Secondary endpoints were as follows: absolute change in mRSS and the total score on the St. George’s Respiratory Questionnaire (SGRQ). A total of 576 patients, the biggest trial on SSc ever, were enrolled: 52% of patients were dcSSc and 48% were receiving stable MMF therapy at baseline. Over the study period, the adjusted annual rate of change in FVC was significantly lower in the nintedanib group compared to the placebo group (− 52 mL/year versus − 93 mL/year, p = 0.04). No significant modification was observed in the mRSS or in the SGRQ between the two groups. Of note, as the primary endpoint of the study was the annual rate of decline in FVC, both lcSSc (with low mRSS) and dcSSc patients were included in the study. No subgroup was identified as better responders although the combination of stable mycophenolate mofetil plus nintedanib provided the best scenario for prevention of decline. The most common adverse event reported, experienced by 76% of nintedanib patients, was diarrhoea; however, it was usually mild and easily manageable with transient reduction of nintedanib and/or anti-diarrheic drugs. Nintedanib has been approved for the treatment of SSc-ILD by the FDA in 2019 and EMA in April 2020.

#### Tocilizumab

Given the encouraging results of the phase II trial of tocilizumab in SSc, a phase III, randomized, double-blind, placebo-controlled, multi-centre trial was performed in early (< 60 months) dcSSc [56]. Patients were assigned to either subcutaneously TCZ 162 mg/week or placebo for 48 weeks. No background immunosuppression was allowed but patients could receive escape therapy from week 16 if they had a decline in FVC or from week 24 if they had a worsening in the mRSS or other SSc-related complications. The primary endpoint of the study was the change in the mRSS at week 48, secondary endpoints were the change in %predicted FVC at week 48 and time to treatment failure, defined as the time from first study drug treatment to the occurrence of the following SSc-related complications: death, decline in FVC > 10%, increase in mRSS > 20% and mRSS > 5 and other predefined SSc-related complications. A total of 212 SSc patients were enrolled. At week 48, the primary endpoint was not met. The improvement in the mRSS was higher in TCZ patients compared to placebo (− 6.1 versus − 4.4) but it did not reach statistical significance (p = 0.098). Nonetheless, the cumulative distribution of change in %predicted FVC favoured TCZ compared to placebo (− 3.9 versus − 0.6, p = 0.0015) and the mean difference in FVC change from baseline was 167 mL in favour of TCZ. A signal towards a higher time to treatment failure was observed in the TCZ group (hazard ratio: 0.63, p = 0.082). Moreover, at week 48, TCZ was associated with a statistically significant higher median ACR CRISS score compared to PBO (0.89 versus 0.25, p = 0.023). Adverse events and serious adverse events were similar between the two groups. The sponsor will not move forward tocilizumab, but the good safety profile, the trend on skin outcomes and the stimulating lung preservation may open the door to further developments using other anti-IL6 agents or similar strategies. Table 2 summarizes Phase 3 trials.

**Table 2:** Targeted therapies of phase 3 studies in SSc patients

Trial drug	Target	Inclusion criteria	Treatment	IS	Endpoints	Duration	Results
Nintedanib	Tyrosine kinase inhibitors (FGF, VEGF, PDGF)	SSc-ILD HRCT ≥ 10% Early (≤ 7 years)	150 mg twice a day	Yes	Annual rate of decline (FVC) mRSS SGRQ	52 weeks	Reduced FVC decline in nintedanib (p = 0.04) No change in mRSS or SGRQ
Tocilizumab	IL-6	Early (≤ 5 years) dcSSc (mRSS ≥ 10)	162 mg sc weekly	No	mRSS FVC	48 weeks	No significant change in mRSS (p = 0.098) Change in FVC favoured TCZ (p = 0.0015)

*IS* immunosuppressive treatment, *SSc* systemic sclerosis, *ILD* interstitial lung disease, *HRCT* high-resolution computed tomography, *FVC* forced vital capacity, *SGRQ* St. George’s Respiratory Questionnaire

### Observational studies

#### Rituximab

A cohort study was published in 2018 including SSc patients from the European Scleroderma Trials and Research network treated with RTX and who were compared to 9575 matched untreated SSc patients and selected using a propensity score matching strategy [57]. The aim of the study was to assess the real-life safety and efficacy profile of RTX in SSc. In total, 254 patients were treated with RTX for several reasons, the main being lung (58%) and skin (32%) involvement. After a median time of 2 years of follow-up, 70% of RTX-treated patients had no side effects. Skin fibrosis improvement (change in mRSS) was more likely observed in RTX group (23 versus 14 events per 100 person-years, odds ratio 2.79, p = 0.002). No significant rates of decrease were observed for %predicted FVC or DLCO. Moreover, a significant higher propensity towards steroid withdrawal or tapering was found in RTX-treated patients (odds ratio 2.34, p < 0.0001) and a significant better lung outcome was found for ILD patients concomitantly treated with mycophenolate mofetil (delta %pFVC 5.22, p = 0.019).

A further 24-week, open-label, randomized, controlled trial was performed to compare the efficacy and safety of RTX compared to intravenous cyclophosphamide (CYC) in early (< 3 years) anti-toposiomerase I-positive dcSSc patients with ILD [58]. No background immunosuppressive treatment was allowed. The primary endpoint was the change in %predicted FVC. Secondary endpoints were as follows: absolute change (in litres) of FVC, mRSS, 6-min walking test, Medsger’s score and new onset or worsening of existing pulmonary hypertension by echocardiography. Sixty patients were randomized to either monthly pulses of CYC 500 mg/m^2^ or RTX 1 gr × 2 infused 2 weeks apart. At 24 weeks, a significant improvement of the %predicted FVC was observed in the RTX group compared to the CYC (61 to 67% versus 59 to 58% respectively, p = 0.003). No significant differences were observed for the other secondary endpoints as the mRSS similarly improved in both the RTX and the CYC group. Serious adverse events were more commonly observed in the CYC group (pneumonia, premature ovarian failure and malignancy).

## Discussion

SSc is characterized by multisystem organ involvement due to the interplay between vascular and immunological and fibrosis pathways [59]. SSc exhibits a remarkable heterogeneity with molecular heterogeneity mirroring the huge clinical one [1]. Recent findings regarding the participation and interaction of several markers and players have led to a better understanding of the pathogenesis of the disease and to the identification of new therapeutic targets. Moreover, improved screening and assessment strategies have promoted earlier detection, stratification and intervention. In addition, immense efforts have led to refine clinical trial design and cohort enrichment strategies, including stimulating biomarkers [60, 61]. There is general consensus about the use of immunosuppressants in early diffuse cutaneous SSc patients [62, 63]. However, the shortcomings of traditional immunosuppressants in SSc, together with the brilliant success of biological DMARDs and small molecule synthetic drugs in inflammatory arthritis, have prompted the investigation of their potential benefits in SSc. Following the paradigms established in inflammatory arthritis, it seems obvious that concurrent or sequential combination therapies will have to be investigated in SSc patients. This is highlighted in the context of SSc by the newly standard of care of upfront combination therapy achieved in pulmonary arterial hypertension [64]. The immediate great challenge thanks to the two recent phase 3 trials, investigating nintedanib [55] and tocilizumab [56], is that we have no evidence to stratify which patients should be treated with anti-fibrotics versus immunosuppression for the SSc-ILD patients. With regard to trial design and selected patients, it seems reasonable to position nintedanib as first-line for patients with established interstitial lung disease and lung fibrotic pattern both as monotherapy and in combination with mycophenolate mofetil. This has been recently further supported by the subgroup analysis of the SENSCIS trial on SSc-ILD patients who were on concomitant mycophenolate mofetil treatment at baseline where it has been shown that a smaller proportion of patients treated with nintedanib versus placebo (29% versus 40% respectively; odds ratio 0,61 [0,37–1,01]) had a decrease in %predicted FVC ≥ 3.3%, which has been estimated to be the minimal clinically important difference for worsening of FVC in SSc-ILD patients [65]. Nonetheless, with the hope of a larger effect to counteract interstitial lung disease, the question of the timing of combination will have to be addressed quickly: generalization of upfront combination? Evaluation first in overlap patients? Restricted to patients failing first-line?

With regard to the other biologics, no firm conclusion can be drawn in the absence of rigorous randomized controlled trials. Nevertheless, it appears in the practice that several SSc patients who fail classical immunosuppressants given at first-line and who may have markers of active inflammation, biologically and or clinically, may receive targeted therapies towards inflammatory cytokines (tocilizumab, abatacept or rituximab). The recent promising patients’ stratification strategies based on autoantibodies status (anti-topoisomerase I versus anti-RNA-polymerase 3 versus anti-centromere etc.) and skin gene profiling offer the opportunity of selecting and identifying the best candidates for each targeted therapy [66]. Hopefully, ongoing or upcoming trials investigating targeted therapies may provide answers and open new avenues in a near future [67].

## Conclusions

We have now an unprecedented arsenal of drugs as in SSc: some new and some already known. We have improved templates for performing clinical trials, and these drugs will be filtered rigorously to weight their risk/benefit ratio. There is a real hope that effective treatment may be available soon in SSc. Once validated, the next step will be to customize patient-specific therapeutics with the goal to develop precision medicine for SSc.

## Data Availability

The datasets used during the current study are available from the corresponding author upon reasonable request.

## Abbreviations

SSc: Systemic sclerosis
dcSSc: Diffuse cutaneous systemic sclerosis
ILD: Interstitial lung disease
DMARD: Disease-modifying anti-rheumatic drug
CYC: Cyclophosphamide
mRSS: Modified Rodnan skin score
RTX: Rituximab
TCZ: Tocilizumab
MMF: Mycophenolate mofetil
MTX: Methotrexate
POM: Pomalidomide
FVC: Forced vital capacity
PBO: Placebo
IS: Immunosuppressive treatment
HRCT: High-resolution computed tomography
FDA: Food and Drug Administration
EMA: European Medical Agency
PRISMA: Preferred Reporting Items for Systematic Reviews and Meta-Analyses
AE: Adverse events
RoBANS: Risk of Bias Assessment Tool for Non-randomized Studies
SCTC GIT: Scleroderma Clinical Trials Consortium Gastrointestinal Tract
DLCO: Diffusing lung capacity for carbon monoxide
HAQ-DI: Health Assessment Questionnaire – Disability Index
CRISS: Combined Response Index in Systemic Sclerosis
PRO: Patient-reported outcome
SGRQ: St. George’s Respiratory Questionnaire
VEGF: Vascular-endothelial growth factor
PF4: Platelet-factor 4
DAMPS: Damage-associated molecular patterns
TLR4: Toll-like receptor 4
IFNAR: Interferon receptor
JAK: Janus kinase
PPAR: Peroxisome proliferator-activated receptor
LPA: Lysophosphatidic acid receptor
ROS: Reactive oxygen species
TGF: Tissue growth factor
CTGF: Connective tissue growth factor
PDGF: Platelet-derived growth factor
ECM: Extracellular matrix
